# Linear Epidermal Nevus of the Oral Cavity: A Rare Diagnosis

**DOI:** 10.1155/2012/206836

**Published:** 2012-06-28

**Authors:** Mariana Dutra de Cássia Ferreira Santos, Alexandre Scalli Mathias Duarte, Guilherme Machado Carvalho, Alexandre Caixeta Guimarães, Carlos Eduardo Monteiro Zappelini, Ana Cristina Coelho Dal Rio, Maria Elvira Pizzigatti Corrêa, Albina Messias de Almeida Milani Altemani, Ester Maria Danielli Nicola

**Affiliations:** ^1^Department of Otolaryngology, ENT, Head and Neck Surgery, Faculty of Medical Sciences (FCM), University of Campinas (UNICAMP), 13083-888 Campinas, SP, Brazil; ^2^Pathology Department, Faculty of Medical Sciences (FCM), University of Campinas (UNICAMP), 13083-888 Campinas, SP, Brazil; ^3^Stomatology Service, ENT Head & Neck Surgery Department, Faculty of Medical Sciences (FCM), University of Campinas (UNICAMP), 13083-888 Campinas, SP, Brazil

## Abstract

Linear epidermal nevus is an uncommon diagnosis of benign lesions of the oral cavity. It is characterized by a congenital malformation arising from the ectoderm cells, which are arranged according to a typical linear configuration known as Blaschko's lines. We report a case of linear epidermal nevus of oral cavity in a 51-year-old lady or woman. The linear epidermal nevus of the oral cavity, although rare, can be considered a differential diagnosis of oral papillomatosis (OP). The histopathological studies and detailed description are the center of the diagnostic and clinical evolution.

## 1. Introduction

Linearepidermalnevus(LEN) is hamartomas from the embrionary ectoderm and is classified according to its major tissue composition (kerotinocytic, follicular, apocrine, eccrine, or sebaceous) [1]. Inflammatory linear verrucous epidermal nevus (ILVEN) counts for approximately 5% of all epidermal nevus [1–3]. The Blaschko line, described by dermatologist Alfred Blaschko in 1901 [4], represents a typical pattern of several nevoid and other skin lesions both congenital [5] or acquired [6] and does not correspond to any known anatomic vascular, nervous, or lymphatic structures [3].

Oral papillomatosis (OP) are hyperplastic verrucous lesions which differential diagnosis includes squamous lesions, vulgar verruca, condyloma, and even congenital lesions as LEN. The diagnosis is mainly according to a histological study [7–9]. OP onset is often prevented using a tetravalent human papillomavirus (HPV) vaccine. However, since LEN is not an infectious pathology, there is no preventive treatment ever described [6]. There are only a few data regarding cases on congenital unilateral papillomatosis and LEN [7]. This paper describes a LEN case in the oral cavity.

## 2. Case Report

**Figure 1:**
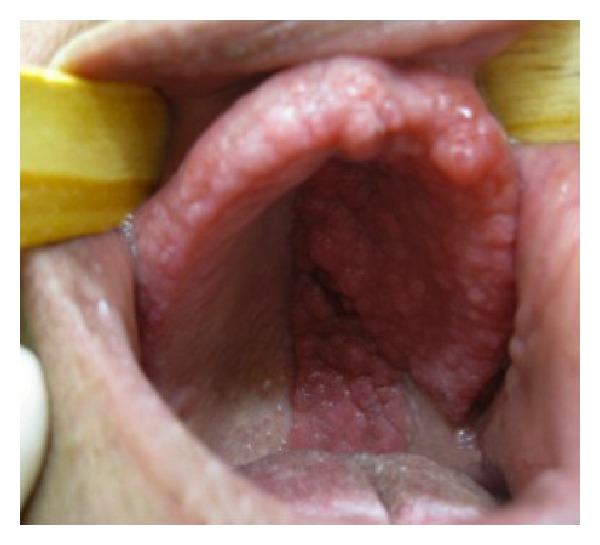
Verrucous lesions without facial midline involvement.

A 51-year-old female patient was attended at the Otorhinolaryngology Clinic of the University of Campinas Teaching Hospital (Campinas, Brazil) on November, 2009, for an oral cavity verrucous lesions evaluation (Figure 1). Patient reported that she had the lesions since birth and they were presenting a painless progressive slow growth in the left hemiplate and bilateral superior labiogingival sulcus. Physical examination showed nasal columella involvement, extending throughout the upper lip, soft and hard palate, and oropharynx with total preservation of the facial midline. 

At the age of 16, patient had a left parotid gland myxoid tumor, being submitted to a partial parotidectomy surgery. After two years, the tumor returned, and she had another surgery. During the new operation, the left facial nerve was injured, resulting in an ipsilateral peripheral facial paralysis. After the procedure, patient received adjuvant radiotherapy.

There are no data regarding the palate lesion pattern before or after these treatments. However, the patient and her family did not reported any lesion change during the subsequently years.

**Figure 2:**
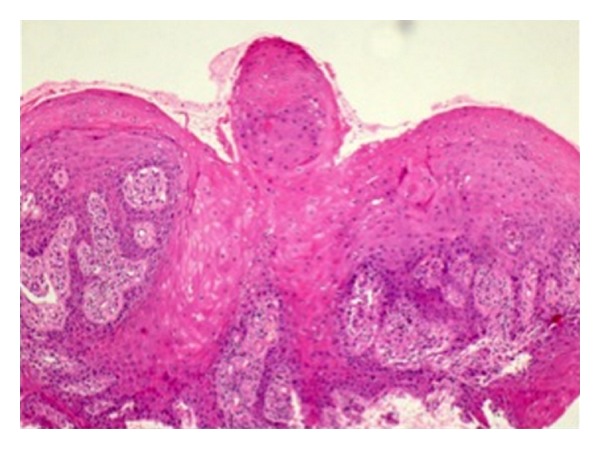
Verrucous lesion extension mainly located on the left side of both soft and hard palate (H and E 200x).

**Figure 3:**
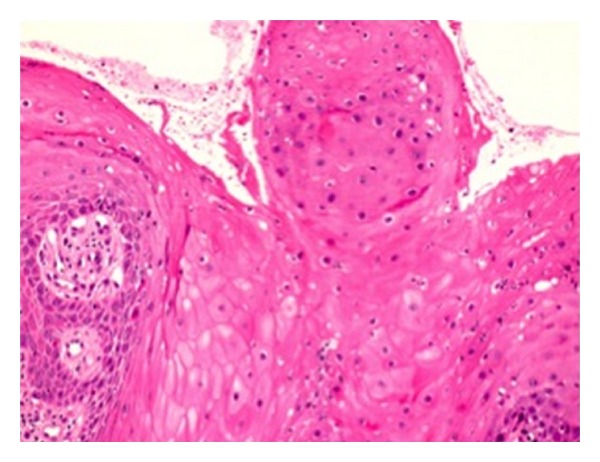
Lesion demonstrates acanthosis and papillomatosis (H and E 400x).

After 11 years since the first procedure, the patient attended at the Otorhinolaryngology Clinic for a palate lesion evaluation. An incisional biopsy was made and the histopathological result reported presence of both gingival e palate tissues with stratified and cornified epithelium, severe hyperplasia, and mild chronic inflammatory infiltration (Figures 2 and 3). The analysis showed no signs of malignancy or external agent infections (e.g., *Candida spp*, HPV). The final diagnosis was oral linearepidermalnevus.

The follow-up decision was to do lesions evaluations every six months. During the first period, the lesions remained the same. Patient is currently under the Otorhinolaryngology Clinic care.

## 3. Discussion

Solomon et al. described in 1968 epidermal nevus lesions combined with noncutaneous organs injuries, which he called epidermal nevus syndrome (ENS) [10, 11]. The most common abnormalities associated to epidermal nevus are found in organs developed from the ectoderm and mesoderm primordial matrix, in special the skeletal system, nervous system, and eyes [11]. It is assumed that LEN is responsible for about 60% of all ENS cases [12].

Oral LEN is a rare disease with only a few cases described in literature [7]. The diagnosis is a match between clinical finds and histopathological analysis [7, 8]. LEN can be classified as a localized or diffuse disease. Diffuse lesions are identified as unilateral or extended bilateral (nevus unius ichthyosis and lateris hystrix, resp.). Localized lesions present a unilateral onset from a specific region [3].

LEN wounds usually affect the vertebral column in a radial pattern injury and the limbs in a longitudinal way. Happle described linear typical manifestations including mucous exclusively involvement of the head and neck regions [3, 4].

Differential diagnosis for LEN is oral papillomatosis [13]. The OP diagnosis is also based on clinical data and histological analysis. Clinical features differ from LEN, mostly regarding onset lesion time, affected areas, evolution pattern, and the fact that it is acquired. Lesion aspect is similar to LEN and can be misleading, except for the preservation of the facial midline [13].

Cutaneous LEN treatment is performed due to an unwanted smell or aesthetic reasons. Bad smell plates can be managed with benzoyl peroxide solutions [1]. For minor obstructive lesions, surgical remove may be considered. Vitamin D analogues can be used for partial regression or retardation of some lesions growth [14]. Lactic acid, salicylic acid, anthralin, intralesional steroid injection, and topical and oral retinoic acid administration have been used with variable degrees of success [1]. Cryotherapy, electrocauterization, dermabrasion, depilation, and surgical remove followed by phenol application have produced inconsistent results [15]. Fibrosis on the ablation-treated area may minimize recurrence risk [1]. Nevus primary thickness is the most determinant factor for aesthetic outcomes. 

In this particular case, lesions characteristics were multiple papules restricted to a hyperplastic mucosa, with facial midline preservation, unilateral, congenital, and without evidence of aggressive behavior. The association among this information and the anatomopathological analysis was pivotal for the final LEN diagnosis.

## 4. Conclusion

Despite LEN rarity, it should always be considered as a differential diagnosis for oral cavity pathologies. The anatomopathological lesions analysis represents a crucial stage in the diagnostic process. Clinical evaluation should always be reported to the pathologist for a better diagnosis.
